# Natural high *p*CO_2_ increases autotrophy in *Anemonia viridis* (Anthozoa) as revealed from stable isotope (C, N) analysis

**DOI:** 10.1038/srep08779

**Published:** 2015-03-05

**Authors:** Rael Horwitz, Esther M. Borell, Ruth Yam, Aldo Shemesh, Maoz Fine

**Affiliations:** 1 The Mina and Everard Goodman Faculty of Life Sciences, Bar-Ilan University, Ramat-Gan 5290002, Israel; 2 Department of Earth and Planetary Sciences, Weizmann Institute of Science, Rehovot 7610001, Israel; 3 The Interuniversity Institute for Marine Sciences, P.O. Box 469, Eilat 8810300, Israel

## Abstract

Contemporary cnidarian-algae symbioses are challenged by increasing CO_2_ concentrations (ocean warming and acidification) affecting organisms' biological performance. We examined the natural variability of carbon and nitrogen isotopes in the symbiotic sea anemone *Anemonia viridis* to investigate dietary shifts (autotrophy/heterotrophy) along a natural *p*CO_2_ gradient at the island of Vulcano, Italy. δ^13^C values for both algal symbionts (*Symbiodinium*) and host tissue of *A. viridis* became significantly lighter with increasing seawater *p*CO_2_. Together with a decrease in the difference between δ^13^C values of both fractions at the higher *p*CO_2_ sites, these results indicate there is a greater net autotrophic input to the *A. viridis* carbon budget under high *p*CO_2_ conditions. δ^15^N values and C/N ratios did not change in *Symbiodinium* and host tissue along the *p*CO_2_ gradient. Additional physiological parameters revealed anemone protein and *Symbiodinium* chlorophyll *a* remained unaltered among sites. *Symbiodinium* density was similar among sites yet their mitotic index increased in anemones under elevated *p*CO_2_. Overall, our findings show that *A. viridis* is characterized by a higher autotrophic/heterotrophic ratio as *p*CO_2_ increases. The unique trophic flexibility of this species may give it a competitive advantage and enable its potential acclimation and ecological success in the future under increased ocean acidification.

Increasing carbon dioxide (CO_2_) emissions drive ongoing ocean acidification (OA) and place marine ecosystems in a vulnerable state^1^. Predictions warn of a further decrease of 0.3–0.5 pH units in oceanic surface water by the end of this century^2^. Natural CO_2_ vents at sub-tropical coastal areas^3^,^4^,^5^ and tropical reefs^6^ serve as natural laboratory locations to study long-term effects of elevated *p*CO_2_ (pH) across many biological and spatial scales. Such a location has been reported in the Levante Bay of Vulcano Island (Italy) in the Mediterranean Sea where many studies have examined physiological adaptations of biota to OA, including seagrass^7^, benthic micro- and macroalgaes^8^,^9^, sea urchins^10^, and sea anemones^11^,^12^. The distinctive characteristics of this location render it a unique environmental setting where the seawater chemistry varies along a *p*CO_2_ gradient of several hundred meters moving away from the venting source. The submarine gas emissions in Levante Bay are characterized by high CO_2_ content volume (>90%) and variable low H_2_S (ranging 0.8 to 2.5% volume)^13^.

A large body of research has focused on the potential impact of OA on reef organisms, particularly scleractinian corals. However, non-calcifying cnidarians such as sea anemones have received less attention^14^. Like many cnidarians, they are mixotrophic organisms, which derive their energy from both photoassimilates translocated from the dinoflagellate symbionts (*Symbiodinium*) and from a variety of external food sources^15^. *Symbiodinium* utilize bicarbonate (HCO_3_^−^), rather than CO_2(aq)_, as the primary source for photosynthesis^16^. Extrinsic sources of carbon for the host include zooplankton and particulate organic carbon (POC)^17^. The two partners that make up the holobiont interact at the basic metabolic level, which includes reciprocal fluxes of energy and nutrient-rich compounds^18^. *Anemonia viridis* Forskål (Cnidaria: Anthozoa), the temperate Mediterranean species chosen for this study, occurs naturally at high densities throughout Levante Bay and harbors the dinoflagellate *Symbiodinium muscatinei* LaJeunesse and Trench (Dinomastigota: Dinophyceae)^12^. Hence it is a powerful comparative model to assess the effects of the changing seawater environment along a natural *p*CO_2_ gradient. Other reports on the response of *A. viridis* near CO_2_ vents discovered changes in their associated microbial communities^19^, reduced dimethylsulfoniopropionate (DMSP) production^12^ and enhanced productivity^3^,^11^.

The purpose of this paper is to investigate dietary changes of *A. viridis* using isotopic compositions, particularly carbon source shifts in the anemone metabolism, in response to high *p*CO_2_/low pH conditions *in situ*. We measured how the natural variability of carbon and nitrogen isotopes in *Symbiodinium* and host tissues of *A. viridis* varies along a natural *p*CO_2_ gradient. This was compared with other key physiological parameters (i.e. total protein concentration; *Symbiodinium* density, mitotic index, and chlorophyll concentration) which were used in the present and in previous studies^11^. Since the δ^13^C and δ^15^N signatures of an organism are related to those of its diet^20^,^21^,^22^,^23^,^24^, our main objective was to estimate the relative contribution of photosynthetic compounds *versus* heterotrophically derived food to the anemone energetic budget (autotrophic/heterotrophic ratio) with increasing seawater *p*CO_2_. This may facilitate better understanding of the environmental fate of cnidarians in a high CO_2_ world.

## Results

**Figure 1:**
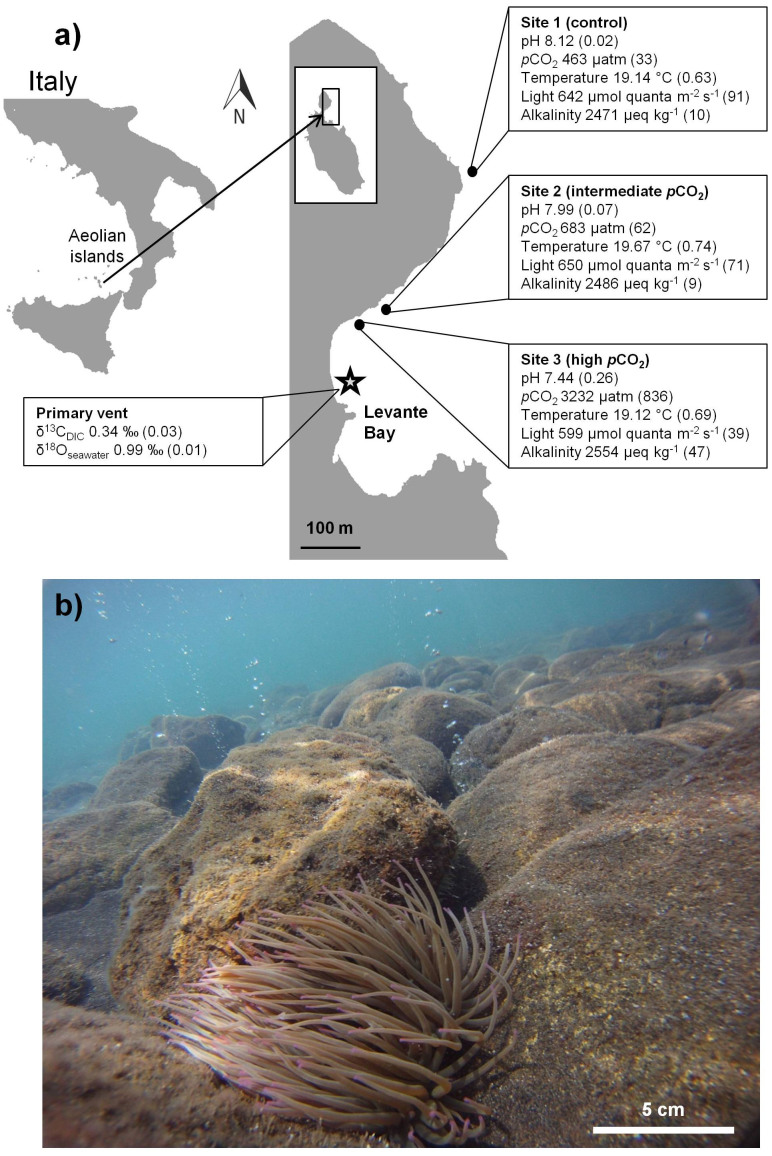
General information on the study sites and the studied organism. (a) Map of the study area with sampling sites 1 (control), 2 (intermediate *p*CO_2_) and 3 (high *p*CO_2_). Boxes show mean values (±SD) of each site for: pH, *p*CO_2_, temperature, light and alkalinity. δ^13^C_DIC_ and δ^18^O_seawater_ (‰) are presented for the primary vent site. The map was created in Adobe Illustrator CS3 (Adobe Systems Inc., San Jose, USA). (b) Image showing *A. viridis* at sampling site 3 (high *p*CO_2_). Photo credit: M. F. (b).

Visual observations made during the course of sampling found anemones at all sampling sites attached to hard substratum at high abundances (of ca. 10–40 anemones m^−2^), consistent with previous findings^11^. Anemones appeared to be healthy with their tentacles fully extended and no visible excess amounts of mucus at the high *p*CO_2_ site (Fig. 1b). Data for seawater pH, *p*CO_2_, TA, temperature and light intensity at all anemone sampling sites is summarized in Figure 1a.

### Total protein, *Symbiodinium* density, mitotic index and chlorophyll concentration

**Figure 2:**
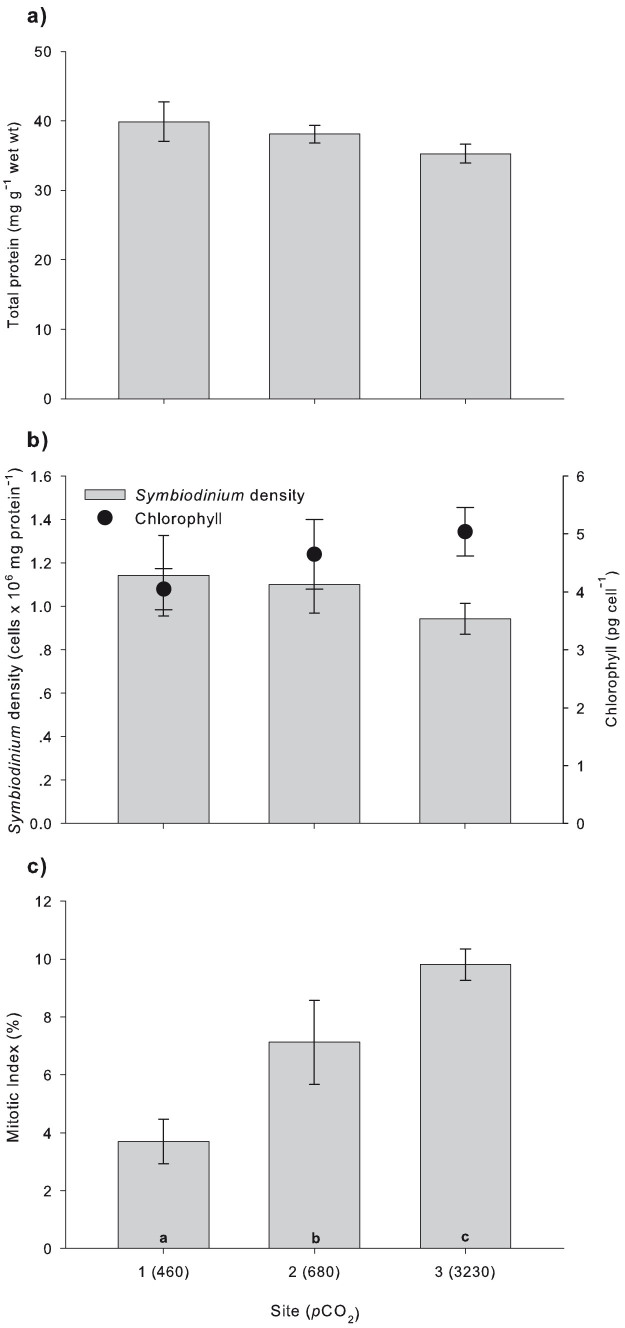
Physiological parameter measurements of *A. viridis* from sites 1 (control), 2 (intermediate *p*CO_2_) and 3 (high *p*CO_2_). (a) Protein concentration (n = 16). (b) *Symbiodinium* density (bars) and chlorophyll concentration (circles) (n = 16). (c) Mitotic index (n = 8). Note that the mean *p*CO_2_ (μatm; Table 1) is given in parentheses for each site. All data represent the mean ± SEM. *Letters* indicate significant differences between sites (Tukey, P < 0.05).

There was no significant difference in anemone protein concentration [1-way ANOVA: *F* (2, 45) = 1.438, P = 0.248] (Fig. 2a), *Symbiodinium* density [1-way ANOVA: *F* (2, 45) = 0.583, P = 0.562] and cell chlorophyll *a* concentration [1-way ANOVA: *F* (2, 45) = 1.125, P = 0.334] between sites (Fig. 2b). Mean protein concentration (mg protein g^−1^ wet wt ± SE) between sites was 37.65 ± 1.12. *Symbiodinium* density (cells mg protein^−1^ ± SE) between sites averaged to 1.06 ± 0.07 × 10^6^ and mean chlorophyll *a* content (pg cell^−1^ ± SE) was 4.57 ± 0.27. The number of dividing *Symbiodinium* cells (MI) was progressively greater in anemones inhabiting the higher *p*CO_2_ sites [1-way ANOVA: *F* (2, 21) = 3.722, P = 0.041], increasing from 3.69 ± 0.76% at the control site to 7.12 ± 1.44% and 9.8 ± 0.54% at the intermediate and high *p*CO_2_ sites, respectively (Fig. 2c).

### Seawater isotopic signature

**Figure 3:**
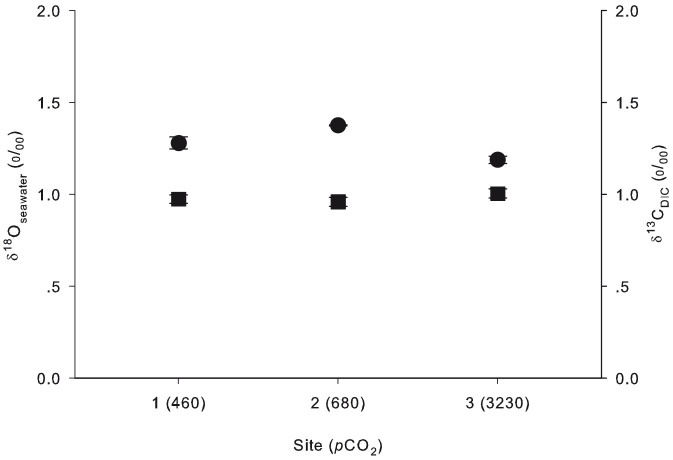
Isotopic measurements of seawater at the sampling sites. δ^13^C_DIC_ (circles) and δ^18^O_seawater_ (squares) (‰) at sites 1 (control), 2 (intermediate *p*CO_2_) and 3 (high *p*CO_2_). Note that the mean *p*CO_2_ (μatm; Table 1) is given in parentheses for each site. All values represent the mean ± SEM (n = 3).

Stable isotope analysis showed constant δ^18^O_seawater_ between all sites, including the primary vent (Kruskal-Wallis ANOVA: df = 3, P = 0.361), with an average of 0.98 ± 0.01‰ (Fig. 3; vent site value not shown). δ^13^C_DIC_ values were similar between sites 1–3, with an average of 1.28 ± 0.05‰ (Fig. 3), although all were significantly heavier compared to the primary vent site (0.34 ± 0.03‰) (Fig. 1a) (Kruskal-Wallis ANOVA: df = 3, P = 0.016).

### δ^13^C variability

**Figure 4:**
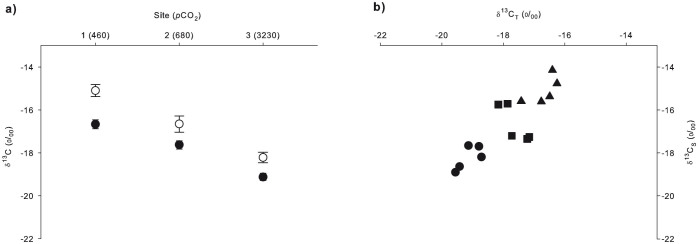
δ^13^C in *A. viridis* from sites 1 (control), 2 (intermediate *p*CO_2_) and 3 (high *p*CO_2_). (a) Mean δ^13^C (‰) values (±SEM; n = 5) of *Symbiodinium* (white circles) and animal tissue (black circles). (b) δ^13^C_T_ vs. δ^13^C_S_ (‰) for individual *A. viridis* specimens from sites 1 (triangles), 2 (squares) and 3 (circles). Note that the mean *p*CO_2_ (μatm; Table 1) is given in parentheses for each sampling site.

δ^13^C values of both animal tissue (δ^13^C_T_) and *Symbiodinium* (δ^13^C_S_) decreased under high *p*CO_2_ conditions (Fig. 4a, b). One-way ANOVA revealed a significant difference in δ^13^C_T_ between all sampling sites [*F* (2, 12) = 42.901, P = 0.000003], with a decrease from −16.66 ± 0.2‰ at the control site to −17.62 ± 0.19‰ and −19.12 ± 0.16‰ at the intermediate and high *p*CO_2_ sites, respectively. δ^13^C_S_ also differed significantly between all sampling sites [1-way ANOVA: *F* (2, 12) = 25.606, P = 0.000047], decreasing from −15.1 ± 0.28‰ at the control site to −16.65 ± 0.37‰ and −18.21 ± 0.24‰ at the intermediate and high *p*CO_2_ sites, respectively. The difference in δ^13^C between the anemone tissue (δ^13^C_T_) and *Symbiodinium* (δ^13^C_S_) at each site was calculated as δ^13^C_S_- δ^13^C_T_ to evaluate changes in autotrophic/heterotrophic ratios. δ^13^C_T_ was considerably lighter than δ^13^C_S_ at all sampling sites with δ^13^C_S_- δ^13^C_T_ reduced with increasing *p*CO_2_ (Fig. 4a). In ambient seawater (control) this difference was relatively large (1.56 ± 0.21‰), while it decreased significantly at the intermediate and high *p*CO_2_ sites (0.96 ± 0.31‰ and 0.9 ± 0.17‰, respectively) [1-way ANOVA: *F* (2, 12) = 5.036, P = 0.026].

### δ^15^N variability and C/N ratios

**Figure 5:**
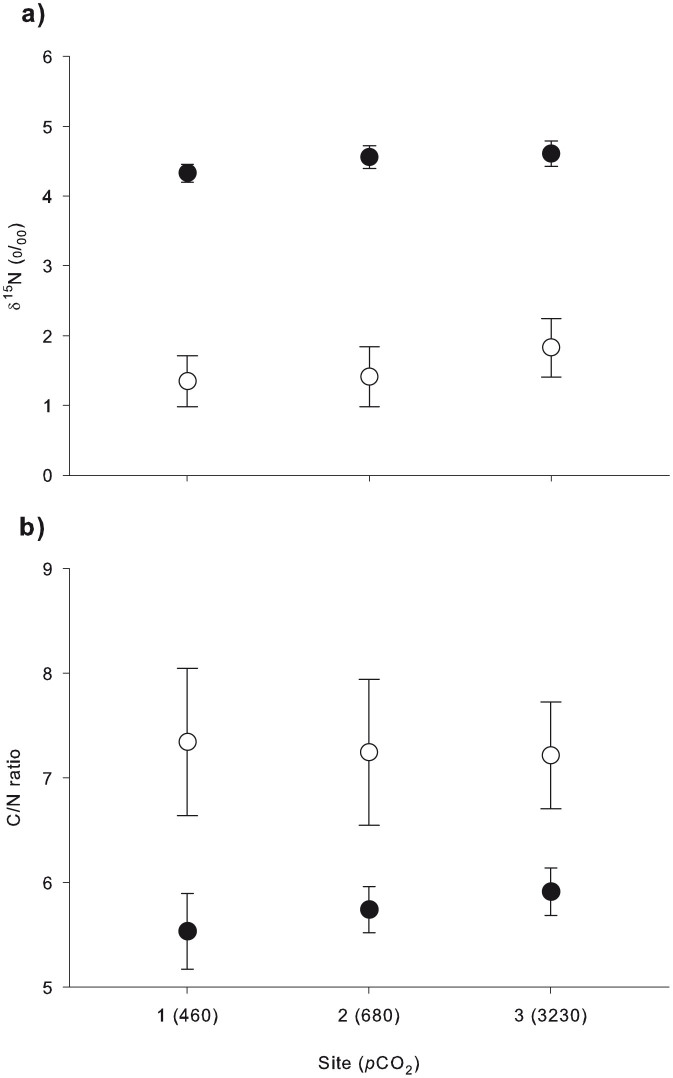
δ^15^N and C/N ratios in *A. viridis* from sites 1 (control), 2 (intermediate *p*CO_2_) and 3 (high *p*CO_2_). Measurements in *Symbiodinium* (white circles) and animal tissue (black circles) of: (a) δ^15^N (‰), and (b) C/N ratio. Note that the mean *p*CO_2_ (μatm; Table 1) is given in parentheses for each site. All values represent the mean ± SEM (n = 5).

There was no significant difference in δ^15^N values of anemone tissue (δ^15^N_T_) [1-way ANOVA: *F* (2, 12) = 0.848, P = 0.452] and *Symbiodinium* (δ^15^N_S_) [1-way ANOVA: *F* (2, 12) = 0.266, P = 0.771] with increasing *p*CO_2_ (Fig. 5a). δ^15^N_T_ was lowest at the control site (4.32 ± 0.12‰) and increased to 4.55 ± 0.16‰ and 4.6 ± 0.18‰ at the intermediate and high *p*CO_2_ sites, respectively. δ^15^N_S_ averaged to 1.34 ± 0.36 at the control site and increased to 1.41 ± 0.42 and 1.82 ± 0.41 at the intermediate and high *p*CO_2_ sites, respectively. δ^15^N_S_ was substantially lighter compared to δ^15^N_T_ at all sampling sites, with an average difference of 2.5 ± 0.23‰ (Fig. 5a). The carbon to nitrogen ratios (C/N) of anemone tissue and *Symbiodinium* did not have any significant differences along the *p*CO_2_ gradient (1-way ANOVAs; *F* (2, 12) = 0.301, P = 0.745 for anemone tissue; *F* (2, 12) = 0.069, P = 0.934 for *Symbiodinium*) (Fig. 5b). The C/N ratio of anemone tissue at the control site was 5.53 ± 0.36 and increased to 5.73 ± 0.21 and 5.91 ± 0.22 at the intermediate and high *p*CO_2_ sites, respectively. The C/N ratio of *Symbiodinium* ranged from 7.34 ± 0.7 at the control site to 7.21 ± 0.51 at the high *p*CO_2_ site.

## Discussion

*A. viridis* collected at all *p*CO_2_ sites lacked any apparent signs of stress (i.e. no mucus, tentacles fully extended; see Fig. 1b). Their general health was further supported by our results for physiological and algal characteristics. Protein concentrations, which are widely accepted as a sensitive indicator for the health of an organism^25^, showed no difference between sampling sites, indicating *A. viridis* was in fact well acclimated to the high seawater *p*CO_2_ (Fig. 2a). In addition, there were no changes in *Symbiodinium* densities and their chlorophyll *a* concentrations along the *p*CO_2_ gradient (Fig. 2b). This is in agreement with observations of the anemone *Anthopleura elegantissima*, following exposure to elevated *p*CO_2_ conditions in a laboratory setting, using the standard algal cell normalization to mg of protein methodology as in the present study^14^. However, *Symbiodinium* densities in *A. viridis* under high *p*CO_2_ conditions nearby the vent at Vulcano have been reported to increase relative to algal densities in anemones at the control site^11^. This discrepancy may be the result of a different methodology (using surface area as a normalization index in the same study^11^) in determining algal cell densities. The handling of anemones greatly influences tentacle contraction, which may have led to inaccuracy in surface area measurement, thereby making the comparison of results difficult.

The substantial increase in dividing algal cells under elevated *p*CO_2_ (MI; Fig. 2c) is in accordance with previous studies reporting high MIs in anemones under high *p*CO_2_^11^,^14^. It is important to note that there was no variation in algal genotype as the anemones from all three sites were found to harbor *Symbiodinium* type A19^12^, excluding the possibility that genetic makeup of the *Symbiodinium* is responsible for the difference. The marked increase in algal division is most likely a direct result of massive CO_2_ input, as *Symbiodinium* in anemones remain carbon limited under normal conditions^11^,^14^,^26^,^27^. Since cnidarians are required to maintain cell-specific densities of their algal symbionts to avoid toxicity from excess oxidative products^28^, the host may initiate either active expulsion of symbionts and/or chemically-signaled arrest of algal reproduction^29^. Here, the high MIs but same algal densities, relative to algal densities at the control site, suggest that the anemones were unable to regulate algal reproduction under the elevated *p*CO_2_ conditions and therefore densities were likely maintained through *Symbiodinium* expulsion. Considering that in addition iron (Fe) is the most important trace element for algal growth^30^, Fe enrichment in the seawater near the vent site^13^,^31^ may have also affected algal proliferation to some extent.

The acidification of seawater close to the venting source arises from the constant gas emissions^13^. In addition to total DIC increasing by 17% at the high *p*CO_2_ site as compared to the control, CO_2(aq)_ increased near the venting source (7-fold increase at the high *p*CO_2_ site; see Table 1). Although the carbonate system still consists mostly of bicarbonate (94%), CO_2(aq)_ increased from less than 1% at the control site to 4% at the high *p*CO_2_ site (Table 1). Nonetheless, the isotopic composition of the inorganic carbon source in this area for the anemones appears to be constant as data shows that δ^13^C_DIC_ does not change between sites (Fig. 3). Consequently, the pronounced and persistent depletion in ^13^C in the tissues of *A. viridis* and its *Symbiodinium* close to the vent cannot be explained by the assimilation of a ^13^C-depleted carbon source. The large increase in *p*CO_2_ in the seawater (Table 1; Fig. 1a) and its availability for *A. viridis* most likely account for the decrease in *A. viridis* δ^13^C values in both *Symbiodinium* and host tissue. The values near the vent (Fig. 4a, b) were well below the lower limit of the range reported previously for both tropical and subtropical sea anemones and *Symbiodinium*^32^,^33^.

δ^13^C_T_ values decreased at the intermediate and high *p*CO_2_ sites to −17.62 ± 0.19‰ and −19.12 ± 0.16‰, respectively, as compared to the control site (−16.66 ± 0.2‰) (Fig. 4a), suggesting an increase in photosynthetically fixed carbon relative to heterotrophically acquired carbon in the host^20^,^34^,^35^. Taking seasonal and regional variability into account, average zooplankton and particulate organic carbon (POC) δ^13^C values reported in the area for surface waters range between −21 and −22‰^36^. We assumed that the availability of these extrinsic carbon sources was constant across all sampling sites in our study, as the relatively short distance between sampling sites (<500 m) and their orientation in Levante Bay towards the open sea renders differences in food availability most unlikely as a factor. Based on mass balance estimation, our calculations show about 5% heterotrophic input to δ^13^C_T_ at the control site (using δ^13^C_T_ = −16.66‰ and δ^13^C_S_ = −15.1‰, assuming δ^13^C_zooplankton/POC_ = −22‰). This is typical of cnidarian-algae symbioses, in which *Symbiodinium* may contribute up to 95% of their photosynthetically-produced carbon to the host^37^. Based on the same assumptions, at the high *p*CO_2_ site the heterotrophic input to δ^13^C_T_ reduced to about 2.5% (using δ^13^C_T_ = −19.12‰ and δ^13^C_S_ = −18.21‰, assuming δ^13^C_zooplankton/POC_ = −22‰), leading to a greater autotrophic input. This observation is also supported by the difference in δ^13^C values between host tissue and *Symbiodinium*, which reflects the relative contribution of heterotrophy and photosynthesis to fixed carbon^20^,^38^. Cnidarian host tissue and *Symbiodinium* stable carbon isotopic values are usually within 2‰ of each other^20^,^39^,^40^. There was a significant reduction in δ^13^C_S_- δ^13^C_T_ with increasing *p*CO_2_ from 1.56 ± 0.21‰ at the control site to 0.96 ± 0.31‰ and 0.9 ± 0.17‰ at the intermediate *p*CO_2_ and high *p*CO_2_ sites, respectively (Fig. 4a). This further indicates an increase in the autotrophic/heterotrophic ratio via translocated autotrophic carbon to the host.

Our results suggest that elevated *p*CO_2_ near the vent promotes carbon isotope fractionation by *Symbiodinium* during photosynthesis, leading to lighter δ^13^C_S_ values. δ^13^C_S_ showed a substantial decrease from −15.1 ± 0.28‰ at the control site to −16.65 ± 0.37‰ and −18.21 ± 0.24‰ at the intermediate and high *p*CO_2_ sites, respectively (Fig. 4a). Many studies have shown that δ^13^C is depleted in marine photosynthetic organisms under elevated *p*CO_2_^41^,^42^,^43^,^44^. Under normal conditions, the majority of *Symbiodinium* carbon requirements (~85%) are met via energy-demanding carbon-concentrating mechanisms (CCMs), whilst the remainder diffuses passively from seawater to the *Symbiodinium* cells^28^. When *p*CO_2_ is elevated, CO_2(aq)_ can replace HCO_3_^−^ as the main carbon source for photosynthesis while energy-consuming CCMs become less important^43^,^45^. Form II ribulose 1,5-bisphosphate carboxylase/oxygenase (form II Rubisco), which is the carboxylating enzyme in *Symbiodinium*^46^, discriminates against ^13^C^47^. Enhanced levels of *p*CO_2_ in the proximity of the vent diffuse to the Rubisco, which favors ^12^C for carbon fixation and ultimately results in a lightning trend of δ^13^C_S_ values. Krief *et al*. (2010) reported the same trend in two species of scleractinian corals after experimental exposure to high *p*CO_2_ in a controlled *p*CO_2_ system. While Krief *et al.* (2010) kept corals under elevated *p*CO_2_ for a period of 14 months, our *in situ* study at the CO_2_ vent site lends insight into a long-term exposure scenario^48^.

δ^15^N_T_ and δ^15^N_S_ values did not change along the *p*CO_2_ gradient, suggesting that the anemones' function and performance reside within normal bounds close to the vent after long-term exposure to acidification conditions (Fig. 5a). Further supporting this concept is the lack of change in C/N ratio between sites (Fig. 5b). The C/N ratio is considered a good proxy for an organism's condition since it reflects the ratio of lipids and carbohydrates to proteins^49^. The apparent absence of preferential accumulation/loss of lipids, carbohydrates or proteins in *A. viridis* in high *p*CO_2_/low pH surroundings indicates therefore that the anemones were well acclimated.

Generally, animals exposed to high *p*CO_2_/low pH have to compensate for acid-base imbalance in intra- and extracellular spaces thereby imposing elevated metabolic costs^50^. A recent study by Laurent *et al*. (2014) demonstrated the high capacity of *A. viridis* to regulate against decreases in internal and external pH, thereby maintaining normal cellular metabolism and physiology^51^. Our results indicate the adaptation and potential resilience of *A. viridis* to acidification conditions, as physiological data (i.e. protein content, *Symbiodinium* density and chlorophyll *a* concentration; Fig. 2a, b), along with δ^15^N values and C/N ratios (Fig. 5a, b), remained unaffected among sites along the *p*CO_2_ gradient. Moreover, the high *p*CO_2_ environment probably stimulated cell division of algal symbionts (Fig. 2c).

We have shown that the anemone host relies more on photosynthetically derived carbon under elevated *p*CO_2_. We propose that *A. viridis* optimizes energy utilization under elevated *p*CO_2_ through an increased autotrophic input, although isotopic data show that heterotrophy is maintained as an additional source of energy/nutrients. These factors may contribute, at least in part, to the increased size and abundance of the *A. viridis* population proximate to the vent site as reported in a previous study^11^. In conclusion, increased autotrophic/heterotrophic ratio may enhance the competitive advantage of symbiotic anemones over other invertebrates and improve their ecological success in benthic communities. These are valuable findings that merit further study for predicting the performance of non-calcifying symbiotic cnidarians in future high-CO_2_ oceans.

## Methods

### Study sites

This study was conducted along the sublittoral in Levante Bay, Vulcano Island (38° 25′ N, 14° 57′ E), part of the Aeolian Island chain, NE Sicily (Fig. 1a) in May 2012. Shallow-water CO_2_ vents create a natural *p*CO_2_/pH gradient along the north-easterly side of the bay, ranging from pH 6.05 to 8.29 at >350 m from the vent site^8^,^13^.

Three sites were selected for animal sampling in accordance with previous studies (see Fig. 1a)^7^,^8^,^11^,^13^. Site 1 (control) was an ambient seawater reference station, located outside the vent area (>400 m); Site 2 (intermediate *p*CO_2_) was ~300 m away from the CO_2_ vents; Site 3 (high *p*CO_2_) was in the proximity of the CO_2_ vents (~260 m). Sampling at the primary vent site (indicated by the star symbol in Fig. 1a) was for collection of seawater samples only.

### Carbonate chemistry and physical measurements

Seawater pH (NBS scale) and temperature were measured at all sites several times a day for 4 days using a pH meter (YSI Professional Plus, Handheld Multiparameter Instrument, USA). Water samples for total alkalinity (TA) analysis were collected from each site, cooled and stored in the dark until analysis. TA was quantified with a Metrohm 862 compact titrosampler^52^. The *p*CO_2_ levels were calculated from salinity ( = 38‰, as reported by Johnson, 2012^53^) and TA and pH_NBS_ measurements using the program CO_2_SYS [Pierrot, D. E., Lewis, E. & Wallace, D. W. R. MS Excel program developed for CO_2_ system calculations. Carbon dioxide information analysis center, Oak Ridge National Laboratory, US Department of Energy, Oak Ridge, TN, USA (2006)], selecting the constants of Mehrbach *et al*. (1973)^54^. Carbonate chemistry parameters are shown in Table 1. Light intensity at each site was measured hourly for 3 consecutive days close to the seabed (1–2 m depth) with HOBO Pendant® Temperature/Light data loggers (Onset, Pocasset, MA, USA). The logged light data were converted from lux to μmol quanta m^2^ s^−1^ (Fig. 1a)^55^.

### Sample collection in the field

#### Anemones

*A. viridis*, a dominant benthic organism in Levante Bay, was prevalent throughout the study area. Sixteen anemones were collected randomly from each site at a depth of 1–2 m and immediately frozen until further analyses. To minimize any confounding responses due to age and/or size all samples were of similar size (oral disc diameter of 2.5–3.5 cm)^56^. Between 5 and 10 tentacles were clipped from each anemone at every site (n = 16). Tentacles were processed for total protein and algal characteristics (i.e., *Symbiodinium* density, chlorophyll *a* concentration and mitotic index) at the sampling site. Samples were weighed (CT 1202, Citizen, accuracy 0.01 g) and homogenized in 0.2 μm sterile filtered seawater (FSW) with an electric homogenizer (DIAX 100 homogenizer Heidolph Instruments GmbH & Co. KG, Schwabach, Germany). The homogenate and all anemones were immediately frozen and then transported on dry ice to the Interuniversity Institute for Marine Sciences (IUI), Israel, where they were stored at −80 °C pending analyses.

#### Seawater

Seawater samples were collected from the four sites for carbon isotopes of dissolved inorganic carbon (DIC; δ^13^C_DIC_) and oxygen isotopic analysis (δ^18^O_seawater_). Triplicate samples for δ^13^C_DIC_ analysis were immediately poisoned upon collection with 60 μl saturated solution of mercuric chloride and stored in 60 ml brown bottles at room temperature until analysis. Triplicate samples for δ^18^O_seawater_ analysis were collected in 50 ml test tubes (Stardest) and stored at room temperature until analysis.

### Total protein, *Symbiodinium* density, mitotic index and chlorophyll concentration

The tissue homogenate of each anemone (n = 16) was further processed and analyzed for measurements of physiological parameters. Total protein analysis was performed by removing 100 μl of the tissue homogenate and sonication on ice with a Branson Sonifier B12 (Branson Sonic Power Co., Danbury, Connecticut, USA) for 20 s. Quantification was done after Bradford (1976) using the Quick Start Bradford Protein Assay Kit and Quick Start Bovine Serum Albumin Standard Set (Bio-Rad Laboratories, Hercules, CA, USA)^57^. Optical density was read at 595 nm using an ELISA reader (Multiskan spectrum, Thermo Fisher Scientific Inc., USA).

For measurement of algal characteristics, 2 ml of homogenate of each sample (n = 16) were centrifuged (5000 rpm at 4°C; 4K15 centrifuge, Sigma) and re-suspended four times in FSW. Re-suspended *Symbiodinium* were used for chlorophyll *a* extraction in acetone (100%) at 4°C in the dark for 24 hours. Concentrations of chlorophyll *a* were measured using spectrophotometry (Ultrospec 2100 pro, GE Bioscience, USA) and calculated using standard equations^58^. Chlorophyll concentration was calculated per *Symbiodinium* cell. *Symbiodinium* densities were quantified from 4 replicate counts using a Neubauer haemocytometer and normalized to protein concentration. Mitotic index (MI) was measured as an indicator of *Symbiodinium* growth and was calculated as a percentage of doublets with a complete cleavage furrow observed per 1000 cells (n = 8 per sampling station)^59^.

### Separation of anemone tissue and *Symbiodinium* for isotope analysis

Sub-samples of 250 mg were excised from the tentacles of each anemone (n = 5 per site) and placed in sterile 15 ml falcon tubes (Stardest). After adding 1 ml 0.2 μm filtered seawater (FSW), an electric homogenizer (DIAX 100 homogenizer Heidolph Instruments GmbH & Co. KG, Schwabach, Germany) was used to homogenize the tissue extract for 2 min. Separation of anemone tissue and *Symbiodinium* was done by the following protocol. The homogenate was centrifuged for 5 min at 5000 rpm (4K15 centrifuge, Sigma) to separate the algae (pellet) and the host tissue (supernatant). Visual inspections revealed no crossover of material between these components, but both were washed carefully.

The host supernatant was homogenized and centrifuged for 10 min at 13,500 rpm (4K15 centrifuge, Sigma, USA), resulting in pelleted host material for analysis. The *Symbiodinium* pellet was then re-suspended in 1 ml FSW, homogenized, and centrifuged for 5 min at 5000 rpm (4K15 centrifuge, Sigma, USA). The procedure was repeated twice in order to remove remaining tissue. All samples were washed with double-distilled water (DDW) to remove any remaining salts. Both the host tissue and *Symbiodinium* samples were dried with a lyophilizer (VirTis, Sentry 2.0, SP Scientific, USA) for 24 h for further isotopic analysis.

### Stable isotope analyses

The isotopic measurements were made at the stable isotopes laboratory in the Department of Earth and Planetary Sciences, the Weizmann Institute of Science, Israel. The oxygen, carbon and nitrogen isotope measurements are reported in the conventional δ-notation.

#### Anemone tissue and Symbiodinium samples

δ^13^C and δ^15^N of 240–270 μg of dried tissue and algae were analyzed using an elemental analyzer (CE 1110) interfaced to the MAT 252 mass spectrometer. Long term precision of working standards for δ^13^C is 0.05‰ and for δ^15^N is 0.1‰ relative to V-PDB and Air respectively (±1σ SD). The carbon to nitrogen ratios (C/N) of anemone tissue and *Symbiodinium* were calculated from simultaneous %C and %N.

#### Seawater samples

δ^18^O_seawater_ was analyzed by equilibrating 0.5 ml of samples with a mixture of 0.5% CO_2_ in He at 25 °C for 24 h. The samples were analyzed on a Gas Bench II connected in-line to a Finigan MAT 252 mass spectrometer. The results are reported relative to VSMOW with 0.08‰ (±1σ SD) long-term precision of the laboratory working standards.

For δ^13^C_DIC_ analysis, 1 ml sea water was injected into vials, flushed with He gas, acidified with 0.15 ml orthophosphoric acid (H_3_PO_4_) and left to react for 24 h at 25 °C. The samples were analyzed on a Gas Bench II and Finigan MAT 252. The results are reported relative to VPDB with 0.08‰ long-term precision (±1σ SD) of the NaHCO_3_ laboratory standard.

### Data analyses

All data was checked for normality using the Kolmogorov-Smirnov test and for homogeneity of variance using Cochran's test. In cases in which homogeneity of variance was achieved, we used one-way ANOVA and a multiple comparison test (Tukey). If homogeneity of variance or normality was not achieved, we used a non-parametric Kruskal-Wallis ANOVA and post-hoc Mann-Whitney U-tests for separation of significant factors. Differences between factors were considered significant for a P value < 0.05. Unless otherwise specified, mean values are presented ± standard error of mean (SEM). All data were analyzed using SPSS version 20 (SPSS IBM, New York, USA).

## Author Contributions

R.H., E.M.B. and M.F. conceived the overall project. R.H. and E.M.B. conducted the field and laboratory work and analysed data. R.Y. and A.S. carried out stable isotope analyses. All authors reviewed and edited the manuscript.

## Figures and Tables

**Table 1:** Carbonate chemistry of seawater at sampling sites 1 (control), 2 (intermediate *p*CO_2_) and 3 (high *p*CO_2_). Parameters were calculated from pH_NBS_, total alkalinity (TA), ambient seawater temperature, and salinity (38‰) using the program CO_2_SYS^54^. All data shown are the mean (±SD). Dissolved inorganic carbon (DIC)

Site	pH_NBS_	TA (μeq kg^−1^)	*p*CO_2_ (μatm)	DIC (μmol kg^−1^)	HC0_3_^−^ (μmol kg^−1^)	CO_3_^2−^ (μmol kg^−1^)	CO_2(aq)_ (μmol kg^−1^)
1. Control	8.12 (0.02)	2554 (47)	463 (33)	2206 (22)	1998 (29)	193 (8)	15 (1)
2. Intermediate *p*CO_2_	7.99 (0.07)	2486 (9)	683 (62)	2287 (46)	2113 (65)	152 (23)	22 (4)
3. High *p*CO_2_	7.44 (0.26)	2501 (20)	3232 (836)	2585 (123)	2430 (93)	50 (25)	105 (53)
